# Progesterone Resistance in Endometriosis: Current Evidence and Putative Mechanisms

**DOI:** 10.3390/ijms24086992

**Published:** 2023-04-10

**Authors:** Ping Zhang, Guoyun Wang

**Affiliations:** 1 Medical Integration and Practice Center, Cheeloo College of Medicine, Shandong University, Jinan 250021, China; 2 Department of Obstetrics and Gynecology, Shandong Provincial Hospital, Shandong University, Jinan 250021, China

**Keywords:** progesterone, progesterone resistance, progesterone receptor, endometriosis

## Abstract

Endometriosis is an estrogen-dependent disease characterized by the growth of endometrial-like tissue outside the uterus. Progestins are currently the most commonly used treatment for endometriosis because of their excellent therapeutic effects and limited side effects. However, progestins have been unsuccessful in some symptomatic patients. The inability of the endometrium to respond properly to progesterone is known as progesterone resistance. An increasing body of evidence suggests the loss of progesterone signaling and the existence of progesterone resistance in endometriosis. The mechanisms of progesterone resistance have received considerable scholarly attention in recent years. Abnormal PGR signaling, chronic inflammation, aberrant gene expression, epigenetic alterations, and environmental toxins are considered potential molecular causes of progesterone resistance in endometriosis. The general objective of this review was to summarize the evidence and mechanisms of progesterone resistance. A deeper understanding of how these mechanisms contribute to progesterone resistance may help develop a novel therapeutic regimen for women with endometriosis by reversing progesterone resistance.

## 1. Introduction

Endometriosis is defined as the presence of endometrial-like tissue [1] that is similar in origin and function in part to the endometrium outside the uterus, with lesions mainly on the pelvic peritoneum but also on the ovaries and rectovaginal septum and more rarely in the pericardium, pleura, and brain. It is a common disease affecting 5–10% of reproductive-age women, characterized by dysmenorrhea, chronic pelvic pain, painful intercourse, infertility, anxiety, and depression, causing a public health problem with a major effect on the quality of life of women and a substantial economic burden [2]. It is well established that estrogen exposure is a major risk factor for this disease, and current treatments primarily focus on suppressing the production and actions of estrogen. Progestins are synthetic compounds that mimic the effects of progesterone by binding to its receptors. The use of progestins and combined oral contraceptives formulated with estrogen and progestin is the most common treatment for endometriosis [3]. Progestins are considered the first-line treatment, especially for the long-term management of endometriosis. These drugs are effective at relieving endometriosis-associated pain and improving the quality of life and are also available in different formulations and routes of administration. Furthermore, their limited side effects make them well-tolerated [4].

However, progestins are unsuccessful in one-third of symptomatic women globally, which is thought to result from the progesterone resistance associated with the disease [2,5]. Progesterone resistance is defined as endometrial progesterone unresponsiveness with subsequent dysregulation of the progesterone signaling pathway and gene networks in the endometrium, which ultimately leads to attenuated progesterone actions [3]. Progesterone resistance has been found in both endometriotic lesions and eutopic endometrium of women with endometriosis [6,7]. In this review, we aim to summarize the evidence supporting progesterone resistance in endometriosis and the currently proposed mechanisms, hoping to facilitate future studies in this field and contribute to the development of a novel therapeutic regimen for women with endometriosis.

## 2. Progesterone and Its Mechanisms of Action

**Figure 1:**
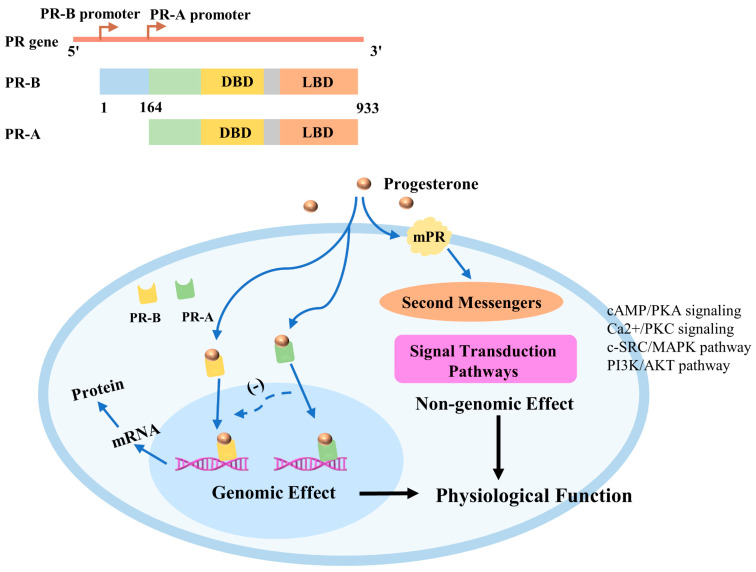
Mechanism of progesterone action. Progesterone effects are primarily mediated by the classical signaling pathway that progesterone receptors bind the ligand and then translocate to the nucleus, where they function as ligand-activated transcription factors and initiate transcription of progesterone-responsive genes. Progesterone also has rapid, non-genomic effects mediated by cell membrane receptors, cytoplasmic PGR, or receptor-independent intracellular signaling cascades. PR-A and PR-B share common structural and functional elements, but PR-A can repress the transcriptional activity of PR-B. DBD: DNA-binding domain; LBD: ligand-binding domain.

Progesterone, a steroid hormone produced by the ovaries, adrenal cortex, and placenta, plays a critical role in the complex regulation of normal female reproductive functions. It is known for its anti-estrogenic effects that suppress endometrial proliferation and the decidualizing effects that drive the transition of the endometrium from the proliferative to the secretory phase. Embryo implantation, pregnancy maintenance, uterine growth, and mammary gland development are also controlled by progesterone [8]. Progestins are synthetic compounds that mimic the effects of progesterone, and both natural progesterone and synthetic progestins are mediated by progesterone receptors (PGR). In response to ligands, PGRs translocate to the nucleus and subsequently bind to gene regulatory regions to regulate the expression of progesterone-responsive genes by directly interacting with specific DNA promoter/enhancer elements or transcription coregulators [9] (Figure 1). Progesterone receptor A (PR-A) and progesterone receptor B (PR-B) are two major progesterone receptors, encoded by the same gene, that share common structural and functional elements. PR-B is a 114 kDa protein, while PR-A is a 94 kDa protein lacking 164 amino acids at the N-terminal [7]. PR-A and PR-B bind to the same steroid hormones with similar binding activities but have different transcriptional activities [10]. PR-B has strong ligand-induced transcriptional activity, whereas PR-A has weak activity but represses the transcriptional activity of PR-B. PR-A and PR-B are considered opposing systems for target cells to control progesterone responsiveness; responsiveness to progesterone is inversely related to the PR-A: PR-B ratio [11]. A PR-B-dominant state promotes progesterone signaling, whereas a PR-A-dominant state decreases progesterone responsiveness.

## 3. Progestins for Endometriosis Treatment

A variety of progestins have been adopted for the treatment of endometriosis, such as dienogest (DNG), norethindrone acetate (NETA), medroxyprogesterone acetate (MPA), cyproterone acetate (CPA), Implanon, and Levonorgestrel-releasing intrauterine system (LNG-IUS). Different compounds can be administered by the oral, intramuscular, subcutaneous, intravaginal, or intrauterine route, exerting systemic and local effects through multiple mechanisms.

### 3.1. Anti-Estrogen

Progestins can inhibit pulsatile GnRH releasing and reduce the secretion of follicle-stimulating hormone (FSH) and luteinizing hormone (LH), which finally leads to the decrease in serum estrogen. In addition, progestins can also down-regulate estrogen receptors (ERs) [12] and restrain the production of local estradiol by activating certain enzymes involved in estrogen metabolism [13]. As a result, progestins limit estrogen-induced lesion growth.

### 3.2. Anti-Inflammation

Activated macrophages and several cytokines are found to be increased in the peritoneal fluid of patients with endometriosis, such as interleukin (IL)-1β, IL-6, IL-8, tumor necrosis factor(TNF)-α, macrophage migration inhibitory factor (MIF), and monocyte chemotactic protein(MCP)-1 [14]. Progestins exert therapeutic effects by impairing the immune-inflammatory response and reversing some alterations of the immune system in endometriosis. It has been demonstrated that they can reduce the secretions of IL-6, IL-8, and MCP-1 and inhibit the cellular proliferation stimulated by TNF-α [15]. For example, dienogest is capable of increasing the activity of natural killer cells in the peritoneal fluid, thus decreasing the number of peritoneal fluid cells and the production of IL-1β by peritoneal macrophages [16].

### 3.3. Anti-Angiogenesis and Inducing Apoptosis

Increasing evidence shows that angiogenesis is a major prerequisite for the initiation and progression of endometriosis. Vascular endothelial growth factor (VEGF) is a potent angiogenic factor involved in physiological and pathological angiogenesis, and elevated levels of VEGF are found in the peritoneal fluid of endometriosis patients [17]. The formation of blood vessels is routinely observed in endometriotic lesions. Dienogest proved to be effective in inhibiting angiogenesis of the ectopic endometrium, with confirmed structural changes in the micro-vessels [18]. In addition, apoptosis is significantly decreased in endometriotic stromal and epithelial cells compared with eutopic endometrial tissues [19]. Progestins can induce apoptosis and atrophy of the endometriotic implants. NETA is reported to show an effect on inducing apoptosis through increased caspase 3/7 activity [20].

### 3.4. Inhibiting Invasion and Oxidative Stress

The establishment of endometriosis is an invasive event that requires degradation of the extracellular matrix, and abundant members of the matrix metalloproteinase (MMP) family play an important role in the process [21]. Progesterone can inhibit the expression of matrix metalloproteinases, thus reducing the capacity of endometrial cells to degrade the extracellular matrix and weaken their invasion and migration ability. Furthermore, it is reported that progestins are able to reduce oxidative stress through the reduction or the abolishment of uterine bleeding [15].

## 4. Evidence of Progesterone Resistance in Endometriosis

### 4.1. Progestin Efficacy and PGR Expression

Progestins are accepted as the major treatment choice in the management of pain and other symptoms associated with endometriosis. Nevertheless, up to a third of women with symptomatic endometriosis do not respond to treatment with progestins and low-dose oral contraceptives, supporting the concept of progesterone resistance. A large number of published studies have revealed the altered expression of PGR in endometriotic lesions and eutopic endometria, especially the loss or attenuation of PR-B expression [7,11,13,22]. Attia et al. [22] reported that peritoneal endometriotic lesion tissue did not express PR-B due to the absence of PR-B transcripts and exhibited lower levels of PR-A compared to the eutopic endometrium. A study by Flores et al. [23] showed that endometriosis patients who did not respond to progestin-based therapies had significantly lower PGR levels than those who did respond, suggesting that PGR status is strongly associated with response to progestin-based therapy. Reduced expression of PGR in endometriosis is perceived as evidence and a cause of progesterone resistance.

### 4.2. PGR Target Genes

Comparative gene expression analysis in the endometrium of women with and without endometriosis showed an attenuated progesterone response in endometriosis [24,25]. The endometrium of women with endometriosis demonstrated dysregulation of numerous progesterone-regulated genes compared to women without endometriosis. These genes include forkhead box O1A (*FOXO1A*), 17b hydroxysteroid dehydrogenase 2 (*HSD17B2*), b-cell lymphoma-2 gene (*Bcl2*), recombinant cytochrome P450 26A1 (*CYP26A1*), homeobox protein hox-A10 (*HOXA10*), and genes that encoding N-acetylglucosamine-6-O-sulfotransferase (GlcNAc6ST) and metallothioneins (MTs). Take *FOXO1A* as an example; *FOXO1A* is involved in cell cycle control and induction of apoptosis by encoding a progesterone-regulated transcription factor, which is induced by decidualization of endometrial stromal cells in response to progesterone [26]. Hence, reduced *FOXO1* expression in the endometrium of women with endometriosis [27] is consistent with a phenotype of attenuated progesterone response. Moreover, genes associated with DNA replication [28], such as proliferating cell nuclear antigen (*PCNA*), a marker of proliferation Ki-67 (*MKI67*), thymidine kinase 1 (*TK1*), cyclin E1 (*CCNE1*), and mitotic arrest deficient-like 1 protein (*MAD2L1*), are downregulated in normal women in response to progesterone. However, previous research has shown that these genes are upregulated in the early secretory endometrium of women with endometriosis [24], suggesting an attenuated progesterone response.

On the other hand, the dysregulation of progesterone target genes was also observed in endometrial stromal cells cultured in vitro from women with endometriosis, even when progesterone levels in the culture medium were well controlled [29], highly supporting the hypothesis that reduced progesterone responsiveness is an intrinsic property in endometriosis instead of resulting from the lower level of circulating or local bioavailable progesterone.

### 4.3. PGR Target Molecules

Quantitative evidence identified alterations in the expression of several progesterone-regulated proteins in the endometrium of women with endometriosis, further substantiating the existence of progesterone resistance in endometriosis. As an immunomodulator regulated by progesterone, glycodelin is important for the immune response during implantation [30], but it is downregulated in patients with endometriosis [24]. MUC-1 and osteopontin are upregulated by progesterone [31] in normal women and act as key factors in embryo attachment, whereas they are downregulated in the secretory endometrium of women with endometriosis [24]. Previous research has recognized the vital role played by the PGR-induced Indian hedgehog—chicken ovalbumin upstream promoter-transcription factor II—WNT family member 4 (IHH-COUPTFII-WNT4) pathway in regulating epithelial proliferation and decidualization during early pregnancy, while the expression levels of all three proteins decrease in the endometrium of endometriosis patients, leading to endometrial non-receptivity [32,33,34].

### 4.4. Progesterone Function

Decidualization denotes the transformation of endometrial stromal fibroblasts into specialized secretory decidual cells and is crucial for embryo implantation and placental development. This process is driven by a postovulatory increase in progesterone levels [35]. As indicated previously, the IHH-COUPTFII-WNT4 pathway is involved in decidualization but is disturbed in endometriosis. Additionally, endometrial stromal fibroblasts obtained from the eutopic endometrium and ectopic endometrium of women with endometriosis demonstrate an impaired ability to decidualize in vitro [36]. Insulin-like growth factor binding protein 1 (IGFBP1) is a classical biochemical marker of decidualization. However, IGFBP-1 shows a nearly two-fold reduction during the window of implantation in the endometrium of women with endometriosis [24], and its secretion by cultured endometrial stromal fibroblasts from women with endometriosis is also reduced [36]. Taken together, these observations strongly suggest an intrinsic abnormality in the progesterone signaling pathway and impaired progesterone effects in endometriosis.

## 5. Mechanisms of Progesterone Resistance in Endometriosis

### 5.1. PGR Deficiency

**Figure 2:**
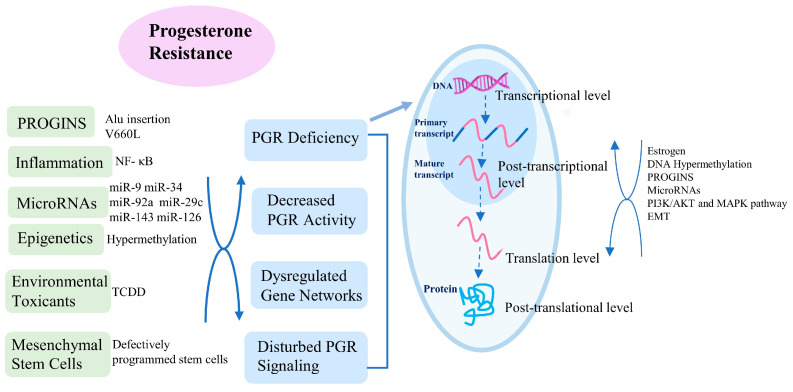
Putative mechanisms of progesterone resistance. Several overlapping but distinct mechanisms are responsible for progesterone resistance. PGR deficiency is considered the most significant mechanism; PGR can be suppressed at several levels in endometriosis.

One of the most significant mechanisms of progesterone resistance is PGR deficiency (Figure 2). Many published studies have assessed the expression of PGR in ectopic endometriosis lesions and eutopic endometrium in women with endometriosis. Most of them did not allow for discrimination between PR-A and PR-B and showed lower PGR levels in ectopic lesion tissue than in eutopic endometrium [37,38,39,40]. Studies that distinguished between PR-A and PR-B tended to find decreased expression of PR-B in endometriosis lesions or eutopic endometrium, whereas reports of PR-A were mixed [41]. In summary, extensive studies have shown that the expression of PR-A and PR-B is generally lower in most types of ectopic lesions, and PR-B deficiency is much more evident. PR-A is the predominant PGR isoform expressed within lesions [13], which is thought to cause an increased PRA: PRB ratio and attenuated progesterone actions. However, Misao et al. observed that PR-B mRNA was expressed at a higher level in some cases of ovarian endometriosis than in eutopic endometrium with a higher PRB: PRA ratio [42]. Although further data collection is required to determine the exact alteration of PGR expression in endometriotic lesions or eutopic endometrium, current studies support PGR deficiency as a crucial mechanism of progesterone resistance.

#### Regulation of Suppressed PGR Expression

- Estrogen

It is generally known that the estrogen response element (ERE) can interact with estrogen receptors (ERs) and initiate a response to estrogen. PR-A promoters contain a half-ERE/Sp1 binding site comprising an ERE half-site and two Sp1 binding sites, which are also involved in estrogen responsiveness and activation of the PR-A promoter. The role of the ERE half-site is to anchor estrogen receptor alpha (ERα) to the half-ERE/Sp1 region and assist in the recruitment of proteins such as transcription factors, which means that ERα stimulates PGR production [43]. Estrogen receptor beta (ERβ) acts as a suppressor of ERα in endometriosis; consequently, excessive levels of ERβ, which is widely recognized in endometriosis [7], may hinder estrogen/ERα-mediated induction of PGR [44,45].

- DNA Hypermethylation

DNA hypermethylation can mediate gene silencing and is an important mechanism of inheritable epigenetic modifications. At the transcriptional level, PGR can be silenced by aberrant DNA methylation of its promoter and first exon. Previous research has suggested that the PR-B promoter is hypermethylated in ectopic endometrium and thus suppresses the expression of PR-B in endometriosis [46]. Moreover, increased methylation of the PR-B gene promoter has only been reported in ectopic endometrial cells but not in eutopic endometrial epithelial cells [46]. The downstream promoter region associated with PR-A transcription remains unmethylated, leading to the disproportional expression of the two PGR isoforms [47]. In addition, Wu et al. [48] reported that DNA methylation enzymes DNMT1, DNMT3A and B are over-expressed in the ectopic endometrium compared with normal control subjects or the eutopic endometrium of women with endometriosis.

- Inflammation

PGR-mediated progesterone actions in the human uterus mainly include progestational and anti-inflammatory actions, which can be withdrawn either by inhibiting PR-B signaling or by decreasing progesterone levels. Declining progesterone levels in the absence of pregnancy lead to an increase in local proinflammatory cytokines, chemokines, and MMPs, which then activate tissue breakdown and menstruation [49]. Nuclear factor (NF)-κB, a major regulator of inflammatory response, is activated by various cytokines increased in the peritoneal fluid of women with endometriosis, such as TNF-α and IL-1β. Moreover, the transcriptional activity of many proinflammatory cytokines, such as IL-1, IL-6, IL-8, TNF-α, MIF, and intercellular cell adhesion molecule-1 (ICAM1), is activated by NF-κB signaling, demonstrating the key role of NF-κB in the inflammatory response in endometriosis [50]. Furthermore, many genes dysregulated in endometriosis are NF-κB target genes, which are related to cell proliferation, adhesion, anti-apoptosis, angiogenesis, oxidative stress, invasion, and inflammation. The NF-κB signaling network has been implicated in endometriosis as an important factor leading to the establishment and maintenance of endometriosis implants [51]. Active NF-κB is reported to be involved in progesterone resistance by suppressing PR-B expression [51]. Ectopic lesions can express increased levels of proinflammatory cytokines, which increases NF-κB expression and decreases PGR expression and progesterone action. Sustained chronic inflammation ultimately contributes to hypermethylation of the PR-B promoter, accounting for permanent progestin resistance. For instance, the proinflammatory cytokine TNF-α was observed to induce hypermethylation of the PR-B promoter in an immortalized endometriotic epithelial cell line [52]. IL-1b is assumed to directly decrease the levels of both PRs isoforms via epigenetic modifications [49].

- Environmental Toxicants

The role of environmental toxicants in the pathogenesis of endometriosis is of significant interest. The 2,3,7,8-tetrachlorodibenzo-p-dioxin (TCDD), a member of the chlorinated aromatic hydrocarbon family, is considered the most toxic dioxin and accumulates in human bodies mainly from contaminated food. A study by Belgians [53] found an increase in TCDD-like toxicants in the blood of women with endometriosis compared to that in disease-free women. Similar findings were noted in an Italian study [54]. TCDD has been shown to disrupt steroid receptor levels, metabolism, and transport, potentially predisposing patients to progesterone resistance and endometriosis. The loss of PGR expression in the endometrium has been observed in TCDD-exposed mice [55]. It is proposed that TCDD-associated PR-B deficiency may be mediated by the local action of toxicant-induced inflammatory cytokines [56]. The biological effects of TCDD are mediated by its binding to the aryl hydrocarbon receptor (AHR), and the ligand-receptor complex then binds to specific dioxin response elements to alter the transcriptional activity of specific genes. Proinflammatory chemokines, such as those regulated upon activation, normal T-cell expressed and secreted (RANTES), can be directly activated by dioxin-AHR complexes in human endometriotic cells [57]. Furthermore, transforming growth factor (TGF) -β2 is a key mediator of progesterone action in the endometrium, which also exhibits anti-inflammatory functions, but it is reported to have low expression in endometrial cells of mice exposed to TCDD [49]. TCDD exposure prevents progesterone-mediated downregulation of MMP expression and blunts its anti-inflammatory responses [58]. Overall, the loss of PGR expression associated with TCDD exposure may result from epigenetic modifications mediated by inflammatory processes.

- Progesterone Receptor Gene Polymorphisms

The progesterone receptor gene (PROGINS) polymorphism consists of the insertion of an Alu element in intron G, a silent point mutation in exon 5 (H770H), and a single amino acid change in exon 4 (V660L) [59]. However, silent mutations in V660L and H770H are not expected to affect PGR transcription and expression [59]. Wieser et al. [60] first described an increased frequency of PROGINS Alu insertion in Caucasian women with endometriosis. Romano et al. reported that PROGINS transcripts are decreased compared with the most common PGR gene transcripts due to the reduced stability of the PROGINS transcripts. It is known that RNA–protein complexes mediate RNA decay by assembling at specific sites on the target transcripts [61]. The Alu element contains a purine-rich region which can be bound by SR proteins, a family of splicing factors, and reduce the stability of the mRNA molecules [61]. Alternatively, pre-mRNA-Alu could be degraded by specific enzymatic mechanisms that Alus transcribed by RNA polymerase III could pair with PROGINS Alu transcribed by RNA polymerase II in the pre-mRNA-Alu as its anti-sense sequence, triggering dsRNA-mediated degradation [59].

- MicroRNAs

MicroRNAs (miRNAs) are non-coding RNA fragments that functionally regulate gene expression by promoting mRNA degradation and inhibiting protein translation. A substantial body of research has shed light on miRNAs as potentially robust biomarkers of endometriosis. Some dysregulated miRNAs are directly involved in disease pathways, and several miRNAs, such as miR-196a, miR-194-3p, miR-29c, and miR-297, are associated with progesterone resistance by modulating the expression of progesterone receptors [62]. In a study by Zhou et al. [63], miR-196a was found to be overexpressed in the eutopic endometrium of patients with endometriosis, whereas its target, PR-B, was found to be significantly decreased. They also proposed that miR-196a downregulates PGR via the ERK/MEK pathway and inhibits decidualization. Pei et al. [64] observed that PGR levels were inhibited by transfection of endometrial stromal cells with an miR-194-3p mimic and were upregulated by miR-194-3p inhibition. miR-29c, which is also overexpressed in the eutopic endometrium of women with endometriosis and baboon models, could downregulate PGR by decreasing FK506-binding protein 4 (FKBP4) levels [65]. miR-297 is also considered a contributor to progesterone resistance to repress the expression of PGR and prevent efficient decidualization in the eutopic endometrium [66].

- Epithelial-to-Mesenchymal Transition

Ma et al. [67] uncovered an interconnection between downregulated PGR expression and the epithelial-to-mesenchymal transition (EMT) in endometriotic lesions. TGF-β-induced EMT, which is marked by elevated levels of Snail family transcriptional repressor 1/2 (SNAI1/2), can cause a significant downregulation of PGR expression in endometriotic epithelial cell lines. Strong expression of SNAI1/2 concurred with weak expression of PGR, not only in vitro but also in endometriotic lesions. They also reported that a low PGR expression was associated with a high N-cadherin expression, which is a hallmark of EMT. These findings strongly suggest a negative correlation between the heterogeneous state of EMT and the suppressed PGR expression in endometriotic lesions.

### 5.2. Decreased PGR Activity

In addition to affecting the expression of progesterone receptors, PROGINS polymorphism contributes to progesterone resistance in other ways. The V660L amino acid substitution, although not affecting PGR expression, could affect the activity of PGR and reduce the action of progestins. It is reported that PGR phosphorylation and subsequent degradation by proteasomal machinery could determine a hyperactivity of the receptor [68]. Romano et al. [59] found incomplete phosphorylation and minor protein degradation of the PROGINS protein variant, which is speculated to impair the activity of PGR. In addition, the PROGINS protein variant showed reduced biological activity. Romano et al. [59] observed less efficient inhibition of proliferation in ovarian cells expressing the PROGINS variant of PR-A. A meta-analysis [69] consisting of 12 studies concluded that the PROGINS polymorphism is related to the risk of endometriosis, and the conferred risk odds ratio in homozygous and recessive models was 1.41–1.43 (*p* = 0.15–0.17). However, this association was observed only in European subjects.

Several proinflammatory cytokines also play vital roles in progesterone resistance by interfering with steroid receptor chaperone proteins or receptor coactivators, such as immunophilin FK506 binding protein 5 (FKBP5) and hydrogen peroxide-inducible clone 5 (HIC-5) [70]. Aghajanova et al. [70] indicated that progesterone resistance in the endometrium of women with endometriosis may partly result from the impaired expression of the PGR coactivator HIC-5, of which aberrant regulation can be attributed to compromised activation of the cAMP/PKA pathway in endometriosis, resulting in impaired PGR functional activity.

### 5.3. Dysregulated Gene Networks

In addition to the aforementioned miRNAs that contribute to an altered PGR expression, there are other miRNAs involved in progesterone resistance. Burney et al. [71] conducted an array-based miRNA profiling in the early secretory endometrium of women with and without endometriosis and found that miR-9 was significantly downregulated in patients with endometriosis compared with women without endometriosis. One predicted target of miR-9 is *BCL2*, a gene encoding an anti-apoptotic protein that is overexpressed in the endometrium of patients with endometriosis. miR-34, which plays a role in the p53-dependent suppression of proliferation, was also downregulated in the early secretory endometrium of patients with endometriosis [71]. Li et al. [72] reported that the highly expressed miR-92a in progesterone-resistant endometriosis is responsible for the low levels of phosphate and tension homolog (*PTEN*), a tumor-suppressing gene that could repress cellular division and promote apoptosis, which eventually leads to higher cell proliferation and resistance to progesterone. Studies have revealed that the overexpression of miR-29c in endometriosis could impair the progesterone response by diminishing the levels of FK506-binding protein 4/52 (FKBP4/52) genes [65]. FKBP4 is a known progesterone-regulated protein responsible for decidualization and is important for embryonic implantation, and FKBP52 is a PGR chaperone protein that governs progesterone actions such as implantation and decidualization in the uterus [73]. These findings can partly explain the failure of progesterone to rapidly halt endometrial proliferation and induce a differentiated state in endometriosis. Warren et al. [74] reported that the increased expression of miR-29c-3p and miR-126-3p, which appear to promote some of the cellular events conducive to endometriosis lesion survival and progression, may be causative factors in the development, progression, and progesterone resistance of endometriosis. However, the exact mechanisms by which miR expression is altered remain to be explored. It has been proposed that the reduced miR expression is the result of altered methylation of miR gene promoters, as treatment with demethylation agents restores normal expression [75].

Previous studies have explored the link between defective epigenetic programming and progesterone resistance. Epigenetic modifications of the transcriptional machinery within endometriotic cells may contribute to the regulation of certain key genes involved in the differentiation process, leading to progesterone resistance [49]. Epigenetic alterations, including DNA and histone methylation and acetylation, and modification of coregulators such as activators, repressors, enhancers, miRs, and other non-coding RNA [49], have the potential to reduce the expression of PGR and other endometrial genes directly or indirectly linked to progesterone, eventually resulting in progesterone unresponsiveness. *HOXA10*, a member of the integral homeobox gene family, is widely known to be associated with endometrial receptivity and progesterone receptor expression. The expression of *HOXA10* is regulated by sex steroids and peaks in the mid-secretory phase [76]. It has been shown that *HOXA10* is aberrantly down-regulated in the endometrium of women with endometriosis during the secretory phase, which is partly due to *HOXA10* promoter hypermethylation and gene-silencing [77].

### 5.4. Disturbed PGR Signaling

The proliferative and anti-apoptotic PI3K/AKT and mitogen-activated protein kinase (MAPK) signaling pathways are hyperactive in endometriosis [78]. Increased levels of phosphorylated AKT and ERK1/2 have been identified in both eutopic and ectopic endometrial tissues from women with endometriosis compared to those in normal endometrium [79]. MAPK and AKT have been confirmed to regulate PGR in breast and endometrial cancer cells; therefore, it is likely that they could contribute to decreased PGR and progesterone resistance in endometriosis in a similar manner [80]. A study by Eaton et al. [81] supported this hypothesis and demonstrated that inhibition of AKT or MEK1/2 could increase PGR levels, decrease proliferation, and promote apoptosis of endometriotic stromal cells and tissues. In addition, increased NOTCH1 activation in endometriotic lesions was correlated with reduced PGR expression [82] and the reduction in Notch signaling was reported to restore PGR and progesterone responsiveness in vitro [83].

The PGR-induced IHH-COUPTFII-WNT4 pathway is of great importance in regulating uterine epithelial proliferation and stromal decidualization. All three proteins decreased in the endometrium of endometriosis patients, as discussed above, indicating disturbed PGR signaling in women with endometriosis. Signal transducer and activator of transcription 3 (STAT3) is a transcription factor that plays an important role in fertility; phosphorylated STAT3 (pSTAT3) is the activated form. pSTAT3, which interacts with PGR signaling to promote implantation and decidualization [84], is significantly higher in the endometrium of both women and non-human primates with endometriosis [85]. B Cell lymphoma 6 (BCL6), a transcriptional gene repressor and a target of STAT3, has been shown to be upregulated in endometriosis. The overexpression of BCL6 appears to result from the phosphorylation of STAT3 in the eutopic endometrium of women with endometriosis [86]. In addition, sirtuin 1 (SIRT1), a histone deacetylase and gene silencer, co-localizes with BCL6 in the nuclei and is upregulated in the endometrium of women and non-human primates with endometriosis. Glioma-associated oncogene homolog 1 (GLI1) is a critical mediator of progesterone activity in the IHH pathway. Both SIRT1 and BCL6 bind to the GLI1 promoter and suppress its expression, leading to a reduction in GLI in women with endometriosis. Further experiments in mice and cell cultures showed that increased BCL6 and SIRT1 expression reduced PGR signaling through the IHH pathway [87].

### 5.5. Mesenchymal Stem Cells

As endometrial mesenchymal stem cells serve as progenitors of endometrial stromal fibroblasts, progesterone resistance in endometriosis may be acquired from these cells. In vitro studies have shown that endometrial mesenchymal stem cells isolated from the eutopic endometrium of women with endometriosis exhibit a failure of decidualization in response to hormone treatment, as well as stromal fibroblasts differentiated from endometrial mesenchymal stem cells, suggesting that progesterone resistance in endometriotic tissue may be inherited from defectively programmed stem cells [88].

## 6. Future Directions

Along with the increase in the incidence rate of endometriosis, the disease has become a growing public health concern worldwide. Nevertheless, a growing body of research suggests the ineffectiveness of progesterone analogs in reducing pain and other symptoms of the disease, which is proposed to result from progesterone resistance associated with endometriosis, appealing to more targeted and effective treatment options for women suffering from endometriosis. To date, therapies that overcome progesterone resistance have been explored in a few studies. The reversal of progesterone resistance, such as re-establishing PGR expression, could inhibit cellular growth and stimulate shedding, thereby providing a novel therapeutic regimen for women with endometriosis. As hypermethylation also contributes to progesterone resistance [46], demethylation agents may be a future research approach for treating endometriosis [89]. Mashayekhi et al. [90] showed that metformin could regulate the expression of dysregulated genes and miRNAs in faulty endometriotic mesenchymal stem cells with impaired differentiation and restore their skewed self-renewal/differentiation balance, making it a promising drug to reverse progesterone resistance and treat endometriosis. Recently, Lin et al. [91] reported that SCM-198 prevents endometriosis in endometriosis mouse models by reversing the low autophagy of endometrial stromal cells via inhibition of the TNF-α-activated aromatase-estrogen-ERα signal and the increase in PR-B expression. In conclusion, further studies in this area have the potential to offer treatment opportunities with superior clinical effectiveness. In the future, there may be more interest in reversing progesterone resistance in the treatment of endometriosis.

## 7. Conclusions

Progesterone resistance is widely viewed as a known pathologic condition in endometriosis as an increasing body of evidence suggests the loss of progesterone signaling in eutopic and ectopic endometrial tissues. The loss of progesterone signaling is a multifactorial process, and the associated mechanisms still remain to be fully understood. PGR-PROGINS, chronic inflammation, aberrant gene expression, epigenetic alterations, and environmental toxins are considered potential molecular causes that further lead to suppressed PGR expression, decreased PGR activity, dysregulated gene networks, and disturbed PGR signaling, which finally results in the state of progesterone resistance. Elucidating the etiology of progesterone resistance will enable the formulation of novel and more effective therapies. In summary, progesterone resistance is a significant issue in endometriosis research and requires more studies to further explore the topic.
